# Banana sRNAome and degradome identify microRNAs functioning in differential responses to temperature stress

**DOI:** 10.1186/s12864-018-5395-1

**Published:** 2019-01-10

**Authors:** Hong Zhu, Yu Zhang, Ruifang Tang, Hongxia Qu, Xuewu Duan, Yueming Jiang

**Affiliations:** 1 Key Laboratory of Plant Resources Conservation and Sustainable Utilization, Guangdong Provincial Key Laboratory of Applied Botany, South China Botanical Garden, Chinese Academy of Sciences, Guangzhou, 510650 China; 2 University of Chinese Academy of Sciences, Beijing, 100049 China

**Keywords:** Banana, sRNAome, Degradome, miRNA, Temperature stress

## Abstract

**Background:**

Temperature stress is a major environmental factor affecting not only plant growth and development, but also fruit postharvest life and quality. MicroRNAs (miRNAs) are a class of non-coding small RNAs that play important roles in various biological processes. Harvested banana fruit can exhibit distinct symptoms in response to different temperature stresses, but the underlying miRNA-mediated regulatory mechanisms remained unknown.

**Results:**

Here, we profiled temperature-responsive miRNAs in banana, using deep sequencing and computational and molecular analyses. In total 113 known miRNAs and 26 novel banana-specific miRNAs were identified. Of these miRNAs, 42 miRNAs were expressed differentially under cold and heat stresses. Degradome sequencing identified 60 target genes regulated by known miRNAs and half of these targets were regulated by 15 temperature-responsive miRNAs. The correlative expression patterns between several miRNAs and their target genes were further validated via qRT-PCR. Our data showed that miR535 and miR156 families may derive from a common ancestor during evolution and jointly play a role in fine-tuning *SPL* gene expression in banana. We also identified the miRNA-triggered phased secondary siRNAs in banana and found miR393-*TIR1*/*AFB* phasiRNA production displaying cold stress-specific enrichment.

**Conclusions:**

Our results provide a foundation for understanding the miRNA-dependent temperature stress response in banana. The characterized correlations between miRNAs and their response to temperature stress could serve as markers in the breeding programs or tools for improving temperature tolerance of banana.

**Electronic supplementary material:**

The online version of this article (10.1186/s12864-018-5395-1) contains supplementary material, which is available to authorized users.

## Background

Banana is a major tropical crop plant worldwide. By far the most important cultivars belong to the triploid AAA group of *Musa acuminata*, commonly referred to as Cavendish group bananas. They accounted for the majority of banana exports, and year-round supply makes them critical for global food security. Bananas are usually refrigerated between 13 °C and 15 °C during storage and transport. At lower temperatures < 12 °C, the peel of bananas gradually turns brown and the ripening permanently stalls. On the other hand, bananas fail to develop an even yellow peel and stay green when ripening at temperatures > 25 °C. There have been reports on the gene regulation of temperature stress response in banana, and some cold resistance-related genes, including a few transcription factors have been characterized [1, 2]. Comparatively, genes associated with fruit heat resistance have rarely been reported. Accumulation of soluble sugars in the peel induced by a high temperature has been proposed as a major factor suppressing chlorophyll degradation, causing the stay-green ripening phenomenon [3]. Overall, the essential factors for the distinct symptoms of banana fruit under temperature stress still remain elusive, and the exploration of underlying mechanism may have practical value for improving banana fruit resistance to temperature stress.

Small regulatory RNAs are major components of eukaryotic transcriptomes. Plants produce many distinct types of DCL/AGO-associated regulatory small RNAs, among which miRNAs and phased siRNAs (phasiRNAs) are the two major types [4, 5]. For more than two decades, miRNAs have been widely studied as important regulatory molecules involved in almost all aspects of the plant life cycle. In plants, the target site usually shows near perfect complementarity to the miRNA sequence, so most target mRNAs are cleaved by RISC, although exceptions have been described where the translation of the mRNA is suppressed without a cleavage [6]. As a subset of plant secondary siRNAs, phasiRNAs can be produced from both protein-coding and non-coding genes. They derive from double-stranded precursors whose synthesis typically depends on an initial miRNA or siRNA interaction and subsequent RDR activity [7, 8]. When there is a single, initiating cleavage event, the dsRNAs all begin at the same nucleotide, so the siRNAs are successively processed from that terminus in a sequential pattern, termed ‘phasing’. Some of the phasiRNAs are capable of acting in *trans* or *cis* [9, 10], so it is intriguing to identify biologically meaningful phasiRNAs from their generating loci (*PHAS* loci) via computational survey and experimental validation. So far, four *TAS* gene families (*TAS1*–*4*) with three specialized initial miRNAs (miR173, miR390 and miR828) have been identified and well characterized in *Arabidopsis* [7, 11, 12]. Recent studies have demonstrated the identification of phasiRNAs in other plants [10, 13, 14].

While initial studies have largely demonstrated the role of miRNAs in morphogenesis and developmental processes in plants, increasing number of studies show that plant miRNAs also target genes involved in stress responses [15]. The first study revealed the up-regulation of miR393, miR397b and miR402 in response to cold, whereas miR319c appeared to be induced by cold but not by dehydration, salinity, or ABA [16]. Recent reports have also demonstrated the role of miRNAs in cold stress response in various plants [17–19]. Also, there are reports on heat-responsive miRNAs in plants [20–22]. Despite these efforts, miRNA investigation in banana has a relatively short history. Although a number of miRNAs have been reported in banana [23–26], so far no banana miRNA sequences have been deposited in the miRNA database - miRBase. Moreover, the role of miRNAs in the temperature stress response of banana fruit remains largely unknown. In this study, we profiled the miRNAs of banana fruit and characterized their expression pattern under cold and heat stress. We comprehensively identified the temperature-responsive miRNAs and their targets, and characterized the miRNA-triggered phasiRNA production in banana. These data provide novel insights into the miRNA-mediated temperature response of banana fruit.

## Results

### Changes in sRNA population of banana under temperature stress

**Fig. 1:**
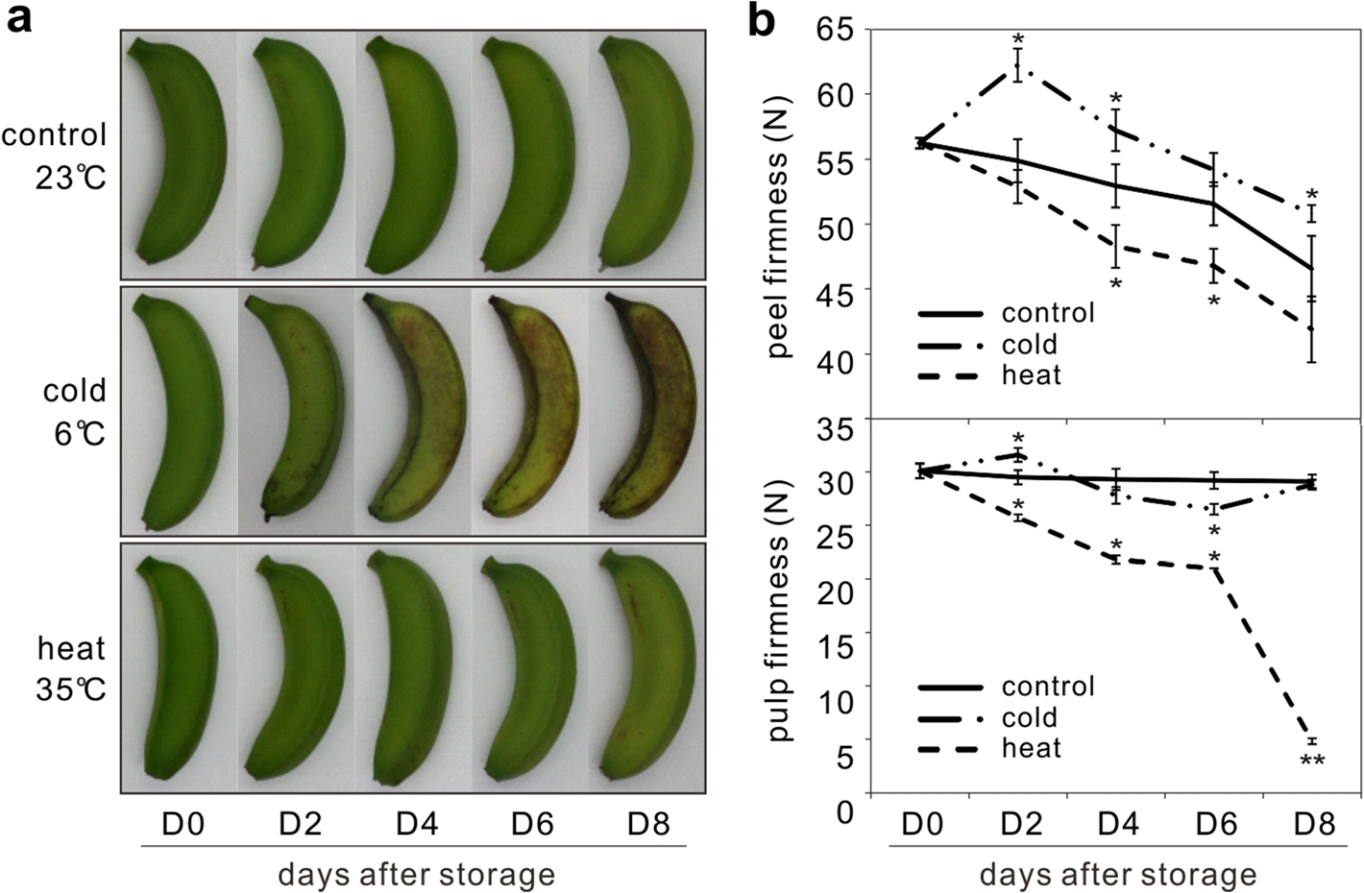
Physiological changes in banana fruit under different temperature stresses. **a** Harvested banana fruit were stored at 23 °C (control), 6 °C (cold) and 35 °C (heat), respectively. Photos were taken every other day from D0 (before storage) to D8 (8 days after storage when symptoms were clearly observed for temperature-stressed fruit). The experiment was conducted twice independently with similar results, and typical photos of the fruit were presented. **b** Changes in fruit firmness under cold and heat treatments. Firmness was measured on unpeeled and peeled fruit fingers for peel and pulp firmness, respectively. Data were presented as the means ± standard errors (*n* = 9) as denoted by the error bars. Asterisks indicated significant differences in expression between control and temperature-stressed samples (*p < 0.05; **p < 0.01)

**Fig. 2:**
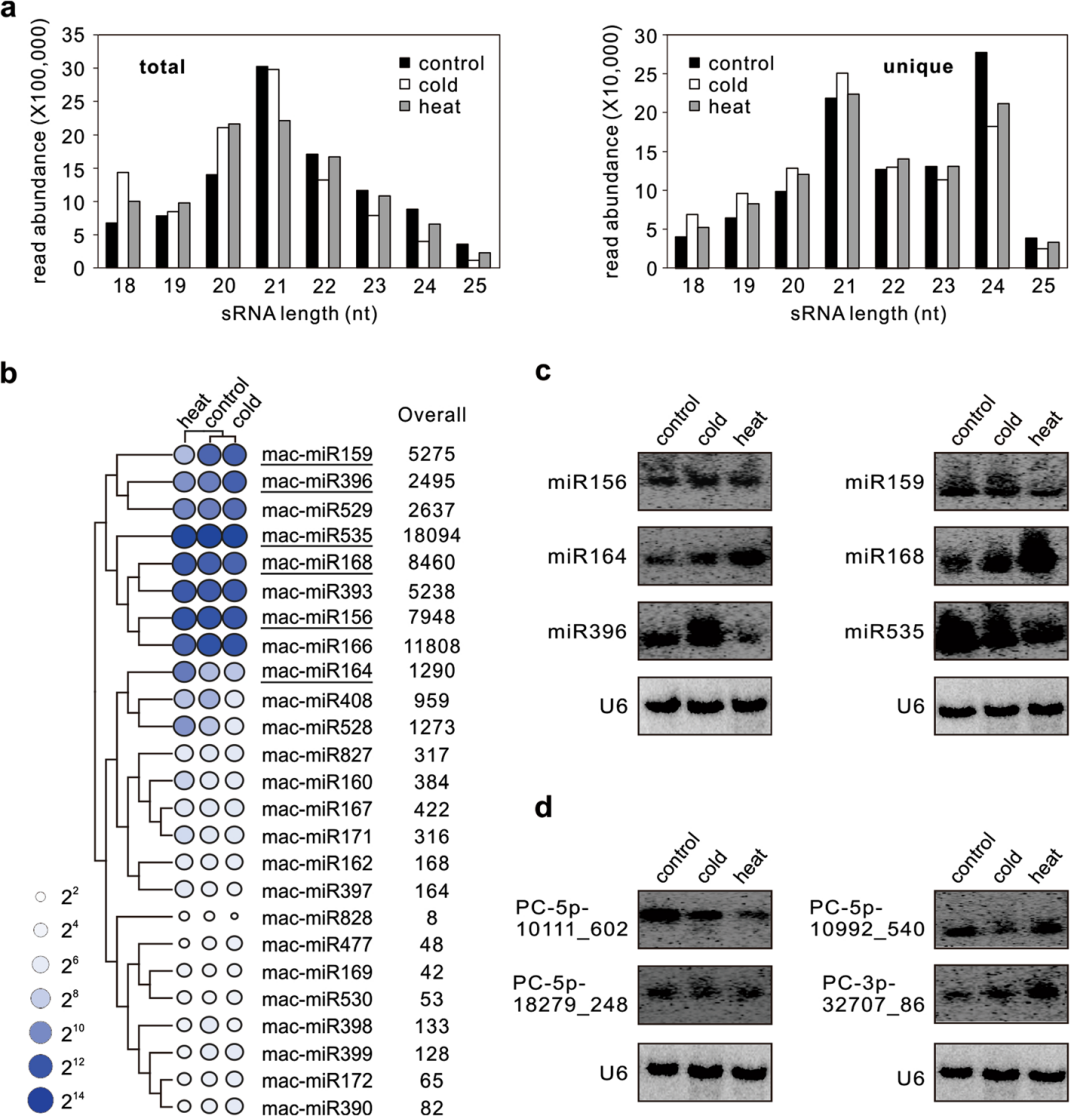
sRNA and miRNA expression profiling in banana. **a** Length distribution of total (left panel) and unique (right panel) sRNA sequences in banana stored at control temperature and subjected to cold and heat treatments. **b** Heat map and overall read abundance of the known miRNAs in the control, cold- and heat-stressed samples. Read abundance was normalized and expressed as reads per million (RPM) of genome-matched reads in each sample, as denoted by color and illustrated at the top-leaf corner of the panel. Known miRNAs selected for validation were underlined. **c**, **d** RNA gel blot analysis of selected known (**c**) and banana-specific (**d**) miRNAs in different samples. Total RNA was isolated from control, cold-stressed and heat-stressed banana peel and probed with end labeled antisense oligonucleotides. U6 served as a loading control. Blotting results from probing and reprobing the same filter were grouped together

Harvested banana fruit were kept at 23 °C (control), 6 °C (cold-stressed) and 35 °C (heat-stressed), respectively for 8 days. Fruit were photographed every other day to monitor the injury symptoms under different temperature stresses (Fig. 1a). Chilling injury of brown spots could be observed as soon as the 2^nd^ day of 6 °C-stored fruit and symptoms intensified as the time progressed. Comparatively, the control and 35 °C-stored fruit showed no change on the appearance (Fig. 1a). The peel firmness of the heat-stressed fruit decreased much faster than the control, and more strikingly a sharp drop of pulp firmness was detected in the heat-stressed fruit after the 6^th^ day. However, cold stress increased the peel firmness especially on the 2^nd^ day while it caused a fluctuation in the pulp firmness (Fig. 1b). Six sRNA libraries of banana peel samples collected from control, cold-stressed and heat-stressed fruit were made for deep sequencing, which yielded 37.3 million high quality reads. Among 3.7 million unique reads, ranging from 18- to 25-nt, ~ 70% were perfectly matched to at least one locus in the banana genome. These reads were used for further analysis (Additional file 1: Table S1). The 20~24-nt sRNAs constituted over 75% of the identified banana sRNAs, and the 21-nt sRNAs was the most abundant class in all samples. The total 21-nt sRNAs were less abundant in the temperature-stressed fruit than in the control, especially for the heat-stressed fruit. In addition, the total 24-nt sRNAs were less abundant in the cold-stressed fruit (Fig. 2a). On the contrary, the unique 21-nt sRNAs were more abundant in the cold-stressed fruit than in the control, and the expression of the unique 24-nt sRNAs was higher than the 21-nt class, but only for the control fruit (Fig. 2a). In the temperature-stressed fruit, both total and unique 18~20-nt sRNAs became more abundant. The increased percentage of these shorter sRNAs may result from the increased RNA degradation products caused by temperature stress. These dynamic changes in the sRNA population clearly showed that cold and heat stresses have differential effects on the sRNA biosynthesis.

### MiRNA profiling in banana

Sequencing reads (21~22-nt) that perfectly mapped to the genome (22 million) were subjected to further analyses for miRNA identification. We identified 113 miRNAs belonging to 25 miRNA families either widely conserved or previously reported. All these miRNAs were collectively referred to as known miRNAs in this study. Expression levels of these known miRNAs, as reflected by normalized reads (RPM), showed a great variation among families in all samples (Additional file 1: Table S2). Most notably, the highest read abundance (~ 18,000 RPM) was detected for miR535 family; the level exceeded most conserved miRNAs. Other high abundance (~ 8000 RPM in total) was detected for miR156, miR166 and miR168 families, which were about 1.5~12 times more than other relatively abundant miRNA families, including miR159, miR393, miR529, miR396, miR164, miR528 and miR408, whose total abundance ranged from 1000 to 5000 RPM (Fig. 2b; Additional file 1: Table S2). While lower expression (between 200 and 500 RPM) was observed for the miR167, miR160, miR171 and miR827 families, their expression level was about twice higher than any of the remaining ten known miRNA families (Fig. 2b; Additional file 1: Table S2). Many known miRNA families comprised multiple species of different mature sequences and distinct length predominance, and showed differential expression among the samples analyzed (Additional file 1: Tables S2).

After excluding sRNA reads homologous to known miRNAs (≤1 mismatch, miRBase 21) and other non-coding sRNAs (Rfam v12.0), the remaining 21~22-nt sRNAs were subjected to novel miRNA identification based on a series of stringent criteria recognized by the research community [27]. A total of 26 miRNA candidates, which had typical hairpin precursor structure and star sequences (miRNA*) found, met the screening criteria (Additional file 1: Table S3), thus were considered novel or banana-specific miRNAs. Among them, 20 belong to the 21-nt class of miRNAs and only six to the 22-nt class (Additional file 1: Table S3). In general, the banana-specific miRNAs were much less abundant compared to the known miRNAs in all samples examined; the highest total read abundance being only ~ 800 RPM, and most yielded levels below 10 RPM (Additional file 1: Table S3).

To validate the sRNA sequencing data, we performed RNA gel blot analysis for selected miRNAs representing known examples in the control and cold/heat-stressed samples (Fig. 2c). We found that the blotting results for most highly expressed known miRNAs were reflective of the relative abundances of sequenced sRNAs. For example, miR164 and miR168 were more accumulated in the heat-stressed sample. Both miR159 and miR396 were induced by cold but greatly repressed by heat stress (Fig. 2c). However, divergence between the two analyses was also observed. Such contradictions between in vivo RNA levels and sequencing results for miRNAs have been previously reported for *Arabidopsis* and grapevine [11, 28]. On the other hand, the low level expression of novel miRNAs was confirmed by RNA gel blot analysis as signal was detectable only for those banana-specific miRNAs with total abundance > 100 RPM (Fig. 2d). As reported above for known miRNAs, RPM values for selected banana-specific miRNAs corresponded to relative signal intensity observed in RNA gel blots in some cases, but divergence was observed as well (Fig. 2d).

### The impact of temperature stress on miRNA expression

**Fig. 3:**
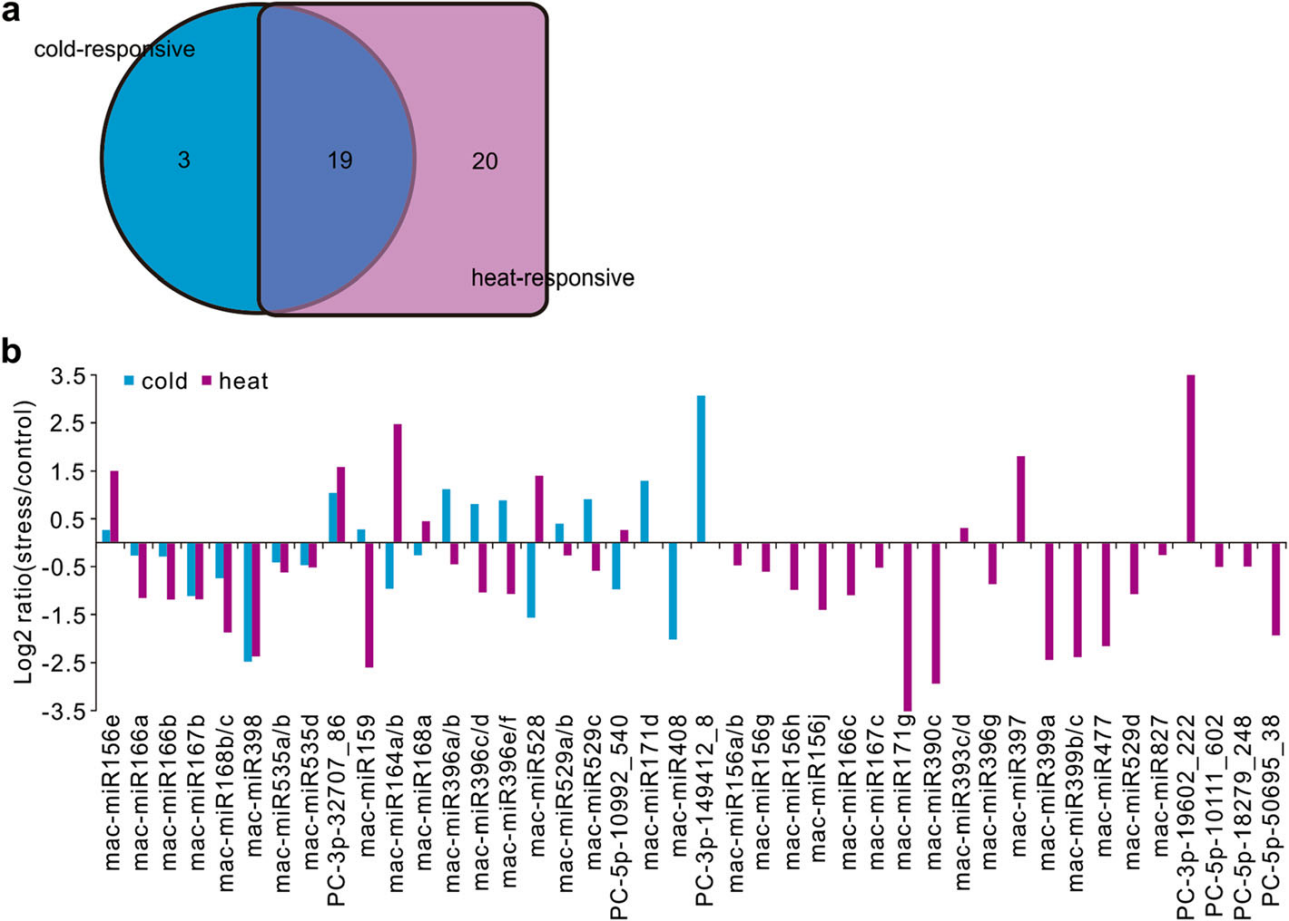
Expression and temperature responsiveness of miRNAs in banana. **a** Venn diagram showing the number of common and specific miRNAs responding to cold or heat stress. The circular and square areas represented cold- and heat-responsive miRNAs, respectively. The overlapping area represented miRNAs responding to both temperature stresses. **b** Fold change of miRNAs in cold- and heat-treated banana fruit on the 5^th^ day of storage, relative to non-treated control. Fold change was calculated on the basis of normalized reads of stressed vs. unstressed samples. Only differentially expressed (*p* < 0.01) miRNAs were shown

To detect the effect of different temperature stresses on banana miRNAs, the expression of miRNAs in banana fruit with cold stress, heat stress and without stress were examined. By comparing the expression level of miRNAs between the control and temperature-stressed fruit (*p* < 0.01), we identified 22 cold-responsive and 39 heat-responsive miRNAs, respectively. Some miRNA families responded to both stressors while a few miRNA families showed stress-specific response (Fig. 3a, Additional file 1: Table S4). About equal numbers of miRNAs were up-regulated and down-regulated by cold, while more miRNAs were down-regulated than up-regulated in the heat-stressed fruit. Among the overlapping group of 19 miRNAs responding to both cold and heat stresses, ten of which showed opposite expression change; the other nine with the same trend (Fig. 3b). Two known miRNAs and one banana-specific miRNA were found to respond only to cold stress. Comparatively, more miRNAs showed obviously altered expression just under heat stress, including 20 known miRNAs and four banana-specific miRNAs. Among these heat-responsive miRNAs, most were down-regulated (Additional file 1: Table S4). We further examined the overlapping group of miRNAs responding to both temperature stresses, and found some miRNAs with targets identified by degradome and having opposite expression patterns between cold and heat stress. For example, mac-miR164a/b targeting a *NAC* gene was down-regulated in the cold-stressed fruit but up-regulated in the heat-stressed fruit. Mac-miR159 targeting a *TCP* gene was induced by cold but repressed by heat. For the three mac-miR396 members that target growth-regulating factor were all up-regulated by cold but down-regulated by heat (Fig. 3b, Additional file 1: Table S4).

### MiRNA target identification and expression validation of temperature-responsive miRNAs and their targets

To identify targets for the banana miRNAs reported here, we performed degradome sequencing to generate a total of 37.4 million short reads representing 5′ ends of uncapped, polyadenylated RNAs. About 70% of the unique reads can be perfectly aligned to the banana transcriptome (https://banana-genome-hub.southgreen.fr/download). These reads were subsequently screened and analyzed with the Cleaveland 3.0 [29]. Based on the miRNA cleavage features on the transcripts, a total of 60 targets falling into five categories (0 to 4) were identified (*p* < 0.05), all for 37 of the 13 known miRNAs or families (Table 1 and Additional file 1: Table S5). Among the 60 targets for the known miRNA families, 90% fell into category 0, which represented the most abundant degradome tags corresponding to the cleavage site and matching cognate transcripts. The number of identified gene targets varied for different miRNAs, ranging from one to eights. For example, miR159 targeted multiple genes encoding TCP transcription factors, miR156 targeted *SQUAMOSA PROMOTER BINDING-LIKE* (*SPL*) genes, and miR167 targeted *AUXIN RESPONSE FACTOR* (*ARF*) genes (Table 1 and Additional file 1: Table S5). Although most of the genes (48 of 60) identified were the conserved targets for these miRNAs across a wide range of plant species, a few of them (12 of 60) had not been reported in other species. For instance, miR156 was also found to target one gene coding for CCR4-NOT transcription factor and miR396 that mainly targets *GROOWTH REGULATING FACTOR* (*GRF*) genes was also found to target one gene encoding metallopeptidase (Table 1 and Additional file 1: Table S5).

**Table 1:** Target of banana known miRNAs (or families)^a^

miRNA	Target	AS^b^	Category^c^	Reads at the cleavage site (tpb)^d^	Target annotation
Conserved targets for known miRNAs					
miR156	Achr6T36010	3	0	722.4	Putative Squamosa promoter-binding-like protein 12
	Achr10T23280	1	0	214.0	Putative Squamosa promoter-binding-like protein 12
	Achr5T21990	1	0	240.8	Putative Squamosa promoter-binding-like protein 12
	Achr10T02970	1	0	989.9	Putative Squamosa promoter-binding-like protein 12
	Achr3T11170	1.5	0	124.9	Putative Squamosa promoter-binding-like protein 17
	Achr5T25090	1.5	0	133.8	Putative Squamosa promoter-binding-like protein 18
	Achr7T22940	1.5	0	2060.1	Putative Squamosa promoter-binding-like protein 16
	Achr8T25550	1.5	0	124.9	Putative Squamosa promoter-binding-like protein 17
miR159	Achr2T12990	3.5	0	693.8	Putative Transcription factor PCF6
	Achr4T22960	3.5	0	292.5	Putative Transcription factor PCF6
	Achr4T31520	3.5	0	194.4	Putative Transcription factor PCF6
	Achr5T02530	3.5	0	881.1	Putative Transcription factor PCF6
	Achr7T01160	3.5	0	881.1	Putative Transcription factor PCF6
	Achr5T06660	3	0	160.5	Putative Transcription factor TCP4
	Achr8T19990	3	0	454.8	Putative Transcription factor TCP4
	Achr10T0102	3	0	481.6	Putative Transcription factor TCP4
miR160	Achr6T18900	1.5	0	321.1	Putative Auxin response factor 22
	Achr9T29480	1.5	0	695.6	Putative Auxin response factor 17
	Achr11T00640	2	0	27,825.2	Auxin response factor 18
	Achr5T18540	2	0	13,029.7	Auxin response factor 18
	Achr5T14630	2	0	4053.4	Auxin response factor 18
	Achr5T03960	2	0	6822.5	Auxin response factor 18
	Achr4T18240	2	0	4080.1	Auxin response factor 18
	Achr8T18930	2	0	3852.7	Auxin response factor 18
miR164	Achr3T23360	3	0	936.4	Putative NAC domain-containing protein
	Achr9T27530	3.5	0	1337.7	no apical meristem protein, putative
	Achr9T26140	2	0	6207.1	NAC domain-containing protein
	Achr9T10210	2	0	20,347.1	NAC domain-containing protein
miR167	AchrUn_randomT06470	3.5	0	5468.7	Auxin response factor 12
	Achr11T25770	4	0	5415.2	Auxin response factor 6
	Achr3T23290	4	0	5281.4	Auxin response factor 17
	Achr5T00590	4	0	4535.0	Auxin response factor 17
	Achr5T26580	4	0	5281.4	Auxin response factor 6
	Achr5T02450	4	0	4454.7	Auxin response factor 6
miR168	Achr3T27070	4	0	1150.5	Protein argonaute 1B
miR171	Achr4T07190	3	0	165,720.2	Putative Scarecrow-like protein 15
	Achr3T29970	3	0	8802.4	Putative Scarecrow-like protein 15
	Achr4T03460	3	0	508.3	Putative Scarecrow-like protein 15
	Achr5T20860	2	0	1551.8	Putative Scarecrow-like protein 6
miR393	Achr6T20580	3	0	4666.5	Transport inhibitor response 1-like protein
	Achr6T16510	3	0	3424.6	Transport inhibitor response 1-like protein
	Achr6T08000	3	0	4882.8	Transport inhibitor response 1-like protein
	Achr9T01140	3	0	4693.3	Transport inhibitor response 1-like protein
	AchrUn_randomT10710	2	0	3917.4	Transport inhibitor response 1-like protein
	Achr10T12000	2	0	3244.0	Transport inhibitor response 1-like protein
miR396	Achr3T15140	2.5	0	615.4	growth regulating factor protein, putative
	Achr7T27480	2.5	0	615.4	growth-regulating factor, putative
miR535	Achr10T02970	4	0	989.9	Putative Squamosa promoter-binding-like protein 12
	Achr4T05720	4	0	695.6	Putative Squamosa promoter-binding-like protein 12
Non-conserved targets for known miRNAs					
miR156	Achr10T05030	3.5	2	240.8	CCR4-NOT transcription factor, putative, expressed
miR164	AchrUn_randomT19770	3	0	20,775.2	Hypothetical protein
	Achr11T02240	4	4	13.4	Probable aquaporin TIP1–1
	Achr8T12920	4	4	13.4	Probable aquaporin TIP1–1
miR396	Achr5T20170	4	1	267.5	Hypothetical protein
	Achr8T26530	2.5	0	615.4	metalloendopeptidase/ metallopeptidase/ zinc ion binding protein, putative
miR528	AchrUn_randomT22730	3.5	0	1172.8	Putative Polyphenol oxidase A1, chloroplastic
	AchrUn_randomT25220	3.5	4	8.9	Putative Polyphenol oxidase A1, chloroplastic
	Achr8T34370	2.5	2	615.4	Polyphenol oxidase, chloroplastic
miR530	Achr2T17590	1	0	2354.4	Hypothetical protein
miR535	Achr4T12470	4	0	668.9	Putative uncharacterized protein
miR828	Achr2T17960	3	0	107.0	Hypothetical protein

^a^ A detailed list was included in Additional file 1: Table S5. ^b^ The alignment score (AS) threshold was set to 4 for known miRNAs. ^c^ Category 0: > 1 raw read at the position, abundance at position is equal to the maximum on the transcript, and there is only one maximum on the transcript. Category 1: > 1 raw read at the position, abundance at position is equal to the maximum on the transcript, and there is more than one maximum position on the transcript. Category 2: > 1 raw read at the position, abundance at position is less than the maximum but higher than the median for the transcript. Category 3: > 1 raw read at the position, abundance at position is equal to or less than the median for the transcript. Category 4: only 1 raw read at the position. ^d^ Reads at the cleavage site were normalized to transcripts per billion (tpb)

**Fig. 4:**
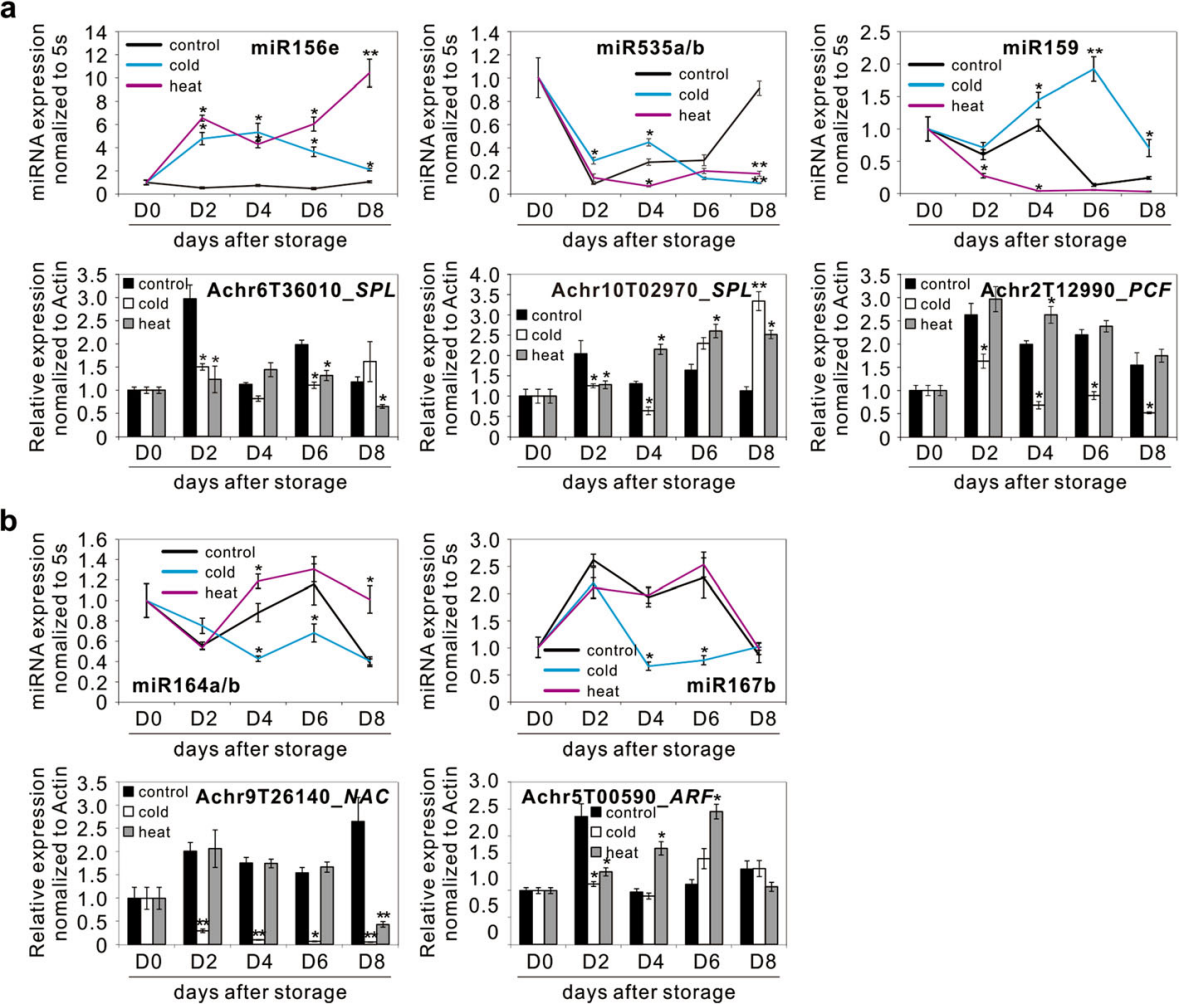
Differential expression of miRNAs and their corresponding targets in control and temperature stressed samples during storage. **a** Anti-correlation pattern of miR156/535-*SPL* and miR159-*TCP* modules. **b** The miR164-*NAC* and miR167-*ARF* modules displaying no obvious correlation. In both (**a**) and (**b**), the top panel showed miRNA accumulation and the bottom panel displayed the corresponding target gene expression. The relative expression was normalized to 5 s rRNA for miRNAs and *Actin* for target genes. Normalized expression in control fruit at day 0 was arbitrarily set to 1. Data were presented as the means ± standard errors (*n* = 3) as denoted by the error bars. Asterisks indicated significant differences in expression between control and temperature-stressed samples (*p < 0.05; **p < 0.01)

We selected several temperature-responsive miRNAs with their corresponding target genes and validated their differential expression in a time-course manner. Some miRNA-target pairs, including miR156/535-*SPL* and miR159-*TCP* modules displayed an inverse correlation (Fig. 4a). However, discrepancies were also observed such as miR164-*NAC* and miR167-*ARF* modules (Fig. 4b), suggesting other levels of regulation may also take effect.

### *SPL* gene family co-targeted by miR156 and miR535 in banana

**Fig. 5:**
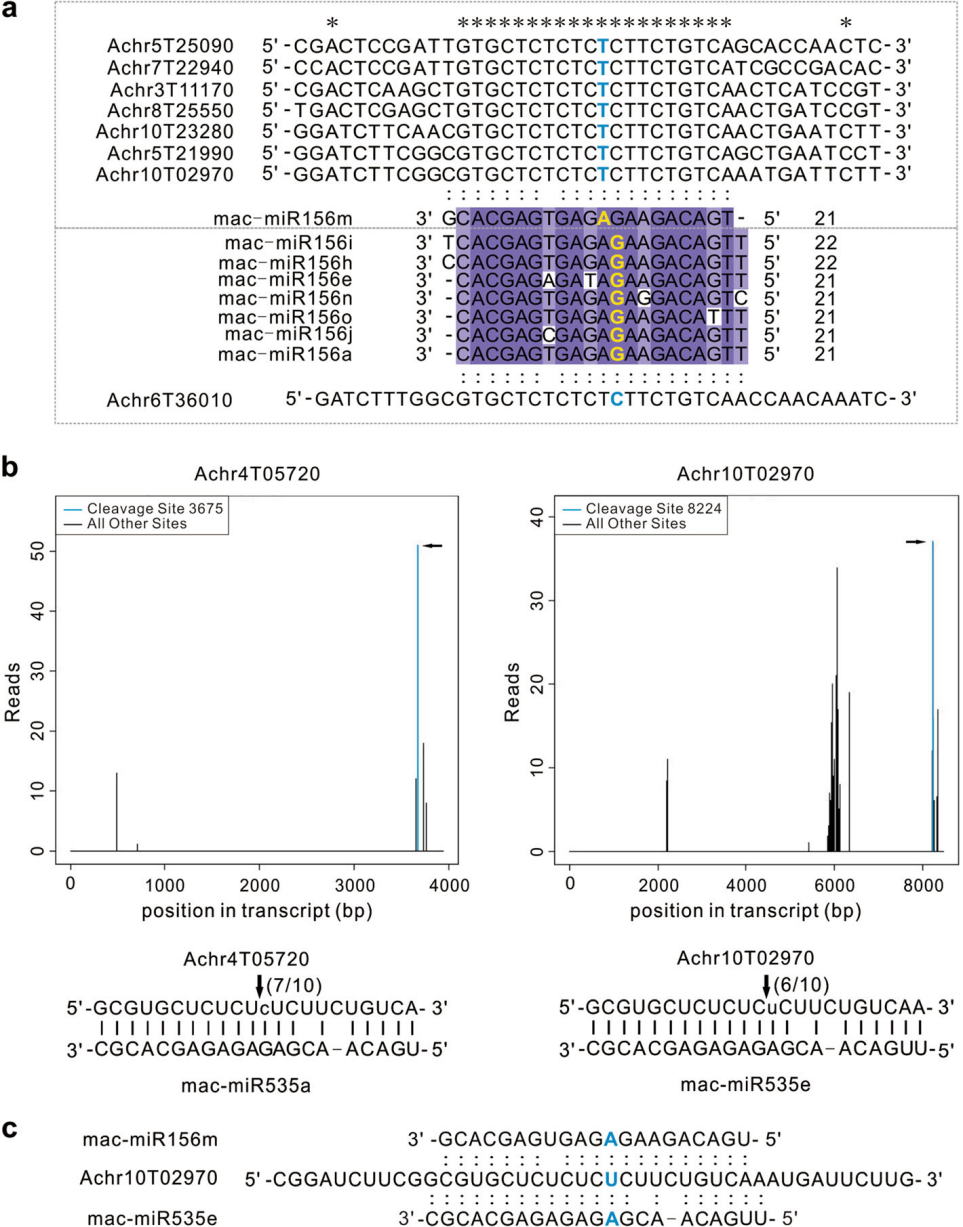
MiR156 and miR535 families co-targeting *SPL* gene family in banana. **a** Sequence alignment of miR156 variants and their targets. The cleavage site detected in the degradome is indicated in blue and yellow letters. **b** Target plot (t-plot) and alignment of validated targets for mac-miR535a/b and mac-miR535e, respectively. The arrow indicates signatures consistent with miRNA-directed cleavage. Cleavage frequency as determined by gene-specific 5′-rapid amplification of cDNA ends (5’-RACE) at the indicated position is shown in parentheses. Primer sequences used for 5’-RACE are provided in Additional file 1: Table S7. **c** Achr10T09270 is co-targeted by mac-miR156m and mac-miR535e. The cleavage sites detected in the degradome are highlighted in blue letters

We found that miR156 displayed a highly conserved target relationship in banana, especially for miR156m variant that was identified to target seven various *SPL* genes, while other miR156 variants collectively targeted one *SPL* gene (Additional file 1: Table S5). Interestingly, there was one nucleotide offset between the cleavage sites of these two groups of *SPL* targets; accordingly miR156m was shorter by 1 nt at the 5′ end (Fig. 5a). In addition to miR156 family, four sequence-specific miRNAs belonging to miR535 family were also identified to target three *SPL* genes, two of which (Achr4T05720, Achr10T02970) was further confirmed by RLM-5’-RACE (Fig. 5b). It was also shown that Achr10T02970 could be cleaved by both miR156m and miR535e at the same location (Fig. 5c). Actually, sequence alignment showed a high similarity among miR156 and miR535 variants and their targets, explaining such a co-targeting phenomenon. While co-targeting the same gene family, different miR156 and miR535 variants exhibited very distinctive expression patterns by temperature stress. For example, mac-miR535a/b was down-regulated by heat stress, whereas mac-miR156e was up-regulated under both stresses, especially heat. Such differential expression patterns were validated by stem-loop qPCR (Fig. 4a). Considering the abundantly and differentially expressed miR535 isoforms, our result suggest that miR535 family may play an equally considerable role, as the well-conserved miR156 family does, in fine tuning *SPL* gene expression in banana.

### Characterization of *PHAS* loci and their triggers in banana

**Fig. 6:**
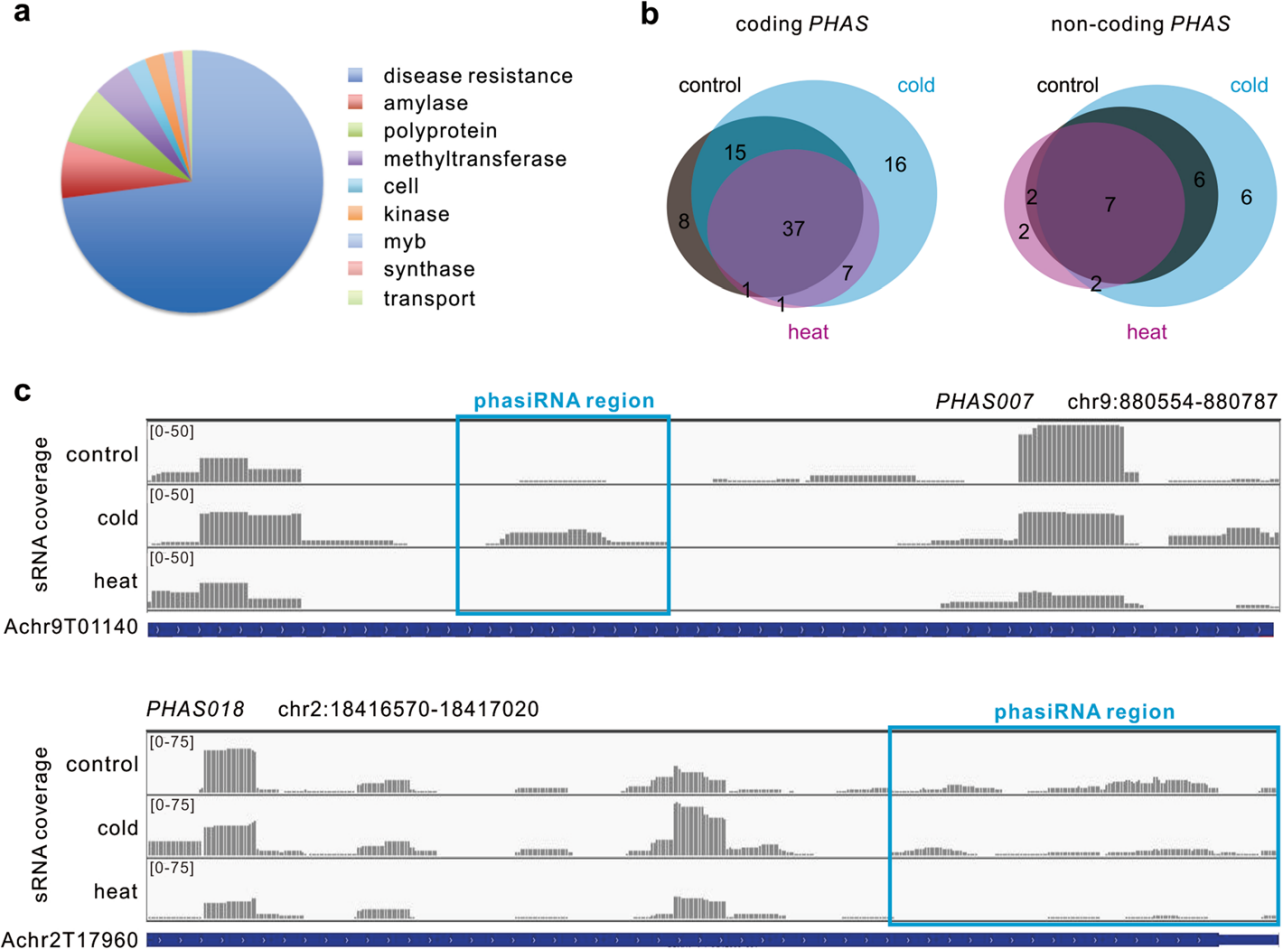
PhasiRNA-generating (*PHAS*) network in banana. **a** All *PHAS* loci were grouped into coding and non-coding genes, and coding *PHAS* loci were further classified based on their annotation, as shown in the pie chart. **b** Venn diagram for coding and non-coding *PHAS* loci differentially accumulated in the control and temperature-stressed samples. **c** Two examples of coding *PHAS* loci displaying differential variations in phasiRNA production in banana upon cold and heat stress. In both panels, each track represented small RNA abundance based on mapping results in the control, cold- and heat-stressed samples. Changes of the phasiRNA accumulation were highlighted in the blue boxes. The gene models from which the sRNA generated were presented below the panels

PhasiRNAs are a major class of sRNAs in plants [9]. They are produced from not only diverse protein-coding genes, including large gene families such as *NB-LRR*s, *PPR*s, and *MYB*s, but also non-coding transcripts [10, 30]. To explore whether phasiRNA pathways are conserved and responsive to temperature stresses in banana, a genome-wide identification of *PHAS* loci was performed. For this analysis, an approach as described in [31] was used. In total, we identified 110 *PHAS* loci (Additional file 1: Table S6), of which 85 loci shown homology to known protein-coding genes, with the remaining 25 loci with no homology found, and thus considered as non-coding loci. Among the coding loci, the majority showed sequence similarity to *NB-LRR* genes (62 or 73%), with other proportions mainly represented by genes coding for alpha-amylase, methyltransferase and polyprotein (Fig. 6a). However, the other two large gene families (*MYB* and *PPR* genes) for generating the majority of phasiRNAs in most eudicots appeared to be rare in banana, where only one gene for MYB protein and none for PPR were identified (Additional file 1: Table S6). Some other coding genes were also found to generate phasiRNAs as well, including genes encoding kinase, starch synthase and transport inhibitor (Additional file 1: Table S6). All non-coding *PHAS* loci were relatively short (< 400 bp), compared to coding *PHAS* loci, suggesting that in banana non-coding *PHAS* loci mainly generate relatively short transcripts.

Next, we determined which miRNAs served as triggers of phasiRNA production for these *PHAS* loci. Six miRNA families were found to function as phasiRNA triggers (Table 2), integrating dagradome data, the miRNAs and the *PHAS* loci. Most miRNA triggers were 22-nt with an initial ‘U’. Several interactions between miRNA triggers and *PHAS* loci were conserved in many other plants, including miR393-*TIR*/*AFB*, miR828-*MYB*, and miR482/miR2118-*NB-LRR*; another three miRNAs, including miR162, miR166 and miR319/miR159, were also identified as triggers. Among them, miR162 and one miR319/miR159 member were also able to trigger phasiRNAs from *NBS*-type genes, but these two were of 21-nt in length, for which the triggering mechanism is not clear. Some miRNA triggers (miR319/miR159, miR393 and miR482/miR2118) could also target non-coding *PHAS* loci, in addition to coding genes (Table 2). Most identified *PHAS* loci (92 or 84%) could not find any known or novel miRNA triggers characterized in this study, indicating a complicated mechanism involved in triggering phasiRNA production. We also found great variations of *PHAS* loci distribution and phasiRNA production in banana fruit upon temperature stress. In general, more *PHAS* loci were found in the cold-stressed fruit while less loci in the heat-stressed fruit, regardless of coding or non-coding *PHAS* loci (Fig. 6b). As examples, two *PHAS* loci exhibited distinct phasiRNAs production in the temperature-stressed samples. The miR393-triggered phasiRNAs were preferentially produced only upon cold stress while the miR828-triggered phasiRNA production was largely abolished upon heat stress (Fig. 6c).

**Table 2:** *PHAS* loci and trigger miRNAs in banana

Trigger miRNA	Sequence	Length (nt)	*PHAS* loci annotation
miR162	UCUAUAAACCUCUGCAUCCGG	21	NBS-type resistance protein RGC5(1e-05)
miR166	UCGUACCAGGCUUCAUUUCCC	21	non-coding
miR319/miR159	UUGGACUGAAGGGAGCUCCUCU	22	non-coding
miR319/miR159	UUGGACUGAAGGGAGCUCCUCU	22	non-coding
miR319/miR159	GAGAGCUUCCUUCAGUCCACU	21	Resistance protein (Fragment)(3e-41)
miR393	UUCCAAAGGGAUCGCAUUGAUC	22	non-coding
miR393	UUCCAAAGGGAUCGCAUUGAUC	22	Transport inhibitor response family protein(3e-37)
miR482/miR2118	UCUUGCCGAUUCCUCCCAUCCC	22	NBS resistance protein (Fragment)(8e-30)
miR482/miR2118	UCUUGCCGAUUCCUCCCAUCCC	22	NBS-LRR disease resistance protein (Fragment)(3e-126)
miR482/miR2118	UCUUGCCGAUUCCUCCCAUCCC	22	NBS-LRR disease resistance protein (Fragment)(2e-79)
miR482/miR2118	UCUUGCCGAUUCCUCCCAUCCC	22	NBS-LRR disease resistance protein (Fragment)(1e-85)
miR482/miR2118	UUUCCAAUACCUCCCAUGCCAA	22	non-coding
miR482/miR2118	UUUCCAAUACCUCCCAUGCCAA	22	non-coding
miR482/miR2118	UUUCCAAUACCUCCCAUGCCAA	22	Os05g0492600 protein(2e-168)
miR482/miR2118	UUUCCGAUUCCUCCCAUCCCUA	22	NBS-type disease resistance protein (Fragment)(4e-84)
miR482/miR2118	UUGCCGAUUCCUCCCAUCCCUA	22	NBS-LRR disease resistance protein (Fragment)(6e-121)
miR482/miR2118	UUGCCGAUUCCUCCCAUCCCUA	22	NBS-LRR resistance protein (Fragment)(3e-76)
miR828	UCUUGCUCAAGUGAGUAUUCCA	22	R2R3 MYB(5e-12)

## Discussion

### MiRNA and sRNA expression in banana

The large-scale miRNA identification via deep sequencing with experimental approaches has been performed in diverse plant species, including many monocots. However, until recently, quite few studies have been done on characterizing miRNAs in banana [23, 24], especially in banana fruit subjected to different temperature stress. In this paper, genome-wide analysis of banana miRNAs were performed with high-throughput sequencing and their response to temperature stress was also analyzed, providing useful information to widen our understanding of miRNAs in monocotyledonous plants. Deep sequencing of the banana sRNA libraries revealed 113 known miRNAs as well as 26 banana-specific miRNAs (Additional file 1: Tables S2 and S3). Most of the conserved miRNA families were identified in our data, but all the banana-specific miRNAs exhibited relatively low expression levels (Additional file 1: Table S3), in consistent with the notion that non-conserved miRNAs are often expressed at a lower level than conserved miRNAs. These miRNAs have not been previously reported in other plants, possibly due to their inappreciable or inducible expression pattern. Deep sequencing and whole-genome data mining enable us to discover probably most of the miRNAs in banana under temperature stress. For all the identified miRNAs, only those showing significant temperature stress responses were further analyzed.

Among the conserved miRNA families, miR156 family appeared to be greatly expanded in banana, having 16 members. MiR535 family was also distinguished because it was the most highly expressed miRNA family in banana, with eight members (Additional file 1: Table S2). In addition, miR535 family was found to co-target *SPL* gene family, together with miR156 family. To date, the origin of most plant *MIRNA*s has not been fully elucidated, although several evolutionary paths have been proposed [31–34]. It has been shown that the origin of plant *MIRNA*s is dependent on the occurrence of various duplications, probably followed by chromosomal rearrangements and loss of duplicated genes, paralleling the coevolution with the mRNA targets [35, 36]. Considering the high sequence homology between miR156 and miR535 variants, these two miRNA families may derive from a common ancestor after duplication events during the evolution. And banana apparently has evolved an expanded miR156/miR535 gene family to conform to their target *SPL* family that comprises a large number of gene members, with the functional importance in banana stress response to be further determined.

PhasiRNAs are a major class of sRNAs in plants. In this study, we identified 110 *PHAS* loci in banana, triggered by six miRNA families. Among the three major protein-coding *PHAS* gene families in eudicots, only *NB-LRR*s yielded abundant phasiRNAs in banana. For other phasiRNA pathways conserved in plants, only miR828-*MYB* and miR393-*TIR1*/*AFB* were identified in banana. On the other hand, some phasiRNA pathways appeared to be newly evolved in banana, although the identification of their trigger miRNAs was unsuccessful (Additional file 1: Table S6). All these results indicate the dynamic feature of *PHAS* pathways in banana during evolution. In modern plants, auxin plays an important role in the regulation of leaf morphology, lateral root growth, and developmental patterning [37, 38], with *ARF* and *TIR* genes being the main components. In banana, profuse *ARF* and *TIR* genes were targeted by miR160/miR167, and miR393, respectively (Additional file 1: Table S5). Moreover, miR393-*TIR1*/*AFB* phasiRNA production displayed a cold stress-specific enrichment (Fig. 6c). However, the well-conserved miR390-*TAS3*-*ARF* pathway appeared to be minimized in banana and the reason for such functional diversification of auxin-related pathways deserves further investigation.

### The temperature-responsive miRNAs in banana

MiRNAs were first revealed to be involved in plant cold response by [16]. Based on a recent review [39], 18 studies in various plant species have confirmed the involvement of miRNAs in response to cold stress, using microarray and sequencing platforms. It has been proposed that response of a particular miRNA to the same cold stress differs depending on the plant species, indicating the divergence of miRNA function during plant evolution. Another explanation would be the tissue-specific miRNA expression pattern that leads to the inconsistency. In those reports, the tested tissues include seedlings, leaves, leaf buds, anther and ovary. Compare to cold-responsive miRNAs, there have been fewer reports of heat-responsive miRNAs in plants, although the specificity of miRNA response to heat stress is also demonstrated [40, 41]. As a typical tropical fruit, banana is very sensitive to cold stress, which usually results in peel pitting, discoloration and abnormal fruit ripening. On the other hand, high temperature stress can result in the ‘stay-green’ ripening disorder of banana fruit. These facts indicate that banana might have evolved differential regulatory mechanisms to react to cold and heat stress. However, information on the role of miRNAs is lacking for harvested banana fruit so far. Exploring the temperature-responsive miRNAs in banana can provide useful information for improving the temperature tolerance and avoidance of ripening disorder of banana fruit. In this study, the expression levels of miRNAs in banana with and without temperature stress treatments were separately compared; around 30% of the miRNAs showed significant changes under temperature stress (Additional file 1: Table S4). Some of these miRNAs have been reported as temperature-responsive in other plants. For example, specific members of miR396 and miR171 families, previously reported as up-regulated by cold in *Arabidopsis* [42], were also found to be differentially induced after cold treatment in banana, indicating that they also conserve in monocots. On the other hand, strong suppression of miR167b, miR398, miR528 and miR408 expression by cold was widely observed in banana. Such down-regulation of miR398 and miR408 partly agreed with previous reports in wheat and grapevine [43], but different from the observation in *Arabidopsis* [42]. In addition, both miR167b and miR528 have never been reported as cold-responsive in plants. Likewise, some previously identified cold-induced conserved miRNAs, such as miR319, miR393 and miR402 in *Arabidopsis* [16], were not found in our data, suggesting that the induction levels of certain miRNAs may be too low to be detected as significant, or they were unaffected by cold in banana. Previous studies have put great emphasis on the cold-induced miRNAs, but cold-repressed miRNAs have comparatively received little attention. Our study showed almost equal numbers of up-regulated and down-regulated miRNAs to cold stress, and these cold-responsive miRNAs had relatively equivalent expression levels (Additional file 1: Table S4). These data suggest that both kinds of regulation for miRNAs were involved in the banana cold response.

In banana, more miRNAs differentially respond to heat stress than to cold stress (Additional file 1: Table S4), suggesting that heat stress might have imposed a wider range of effect on miRNA expression. Moreover, compare to the relatively equal number of down- and up-regulated miRNAs responding to cold, the number of down-regulated miRNAs was almost three times that of up-regulated ones among the identified heat-responsive miRNAs in banana, indicating that overall heat stress tended to inhibit miRNA expression. Several heat-responsive miRNAs identified from banana were also previously reported in other plants. For example, it has been reported that miR156 integrates the response to recurring heat stress with development in *Arabidopsis* through *SPL* TFs [44]. We found that in banana, multiple miR156 variants showed differential response upon heat stress, where miR156e was significantly induced while others got suppressed. Our result indicated that the miR156-*SPL* module might also mediate the heat stress response in banana. Other significantly heat-induced miRNAs included miR164a/b, miR528 and miR397. A similar up-regulation pattern of miR164 has been previously reported in heat-stressed switchgrass [40], but the case was opposite in wheat [41]. On the contrary, a large number of banana miRNAs, including miR159, miR166c, miR171g, miR390c, miR396a-f, miR398, miR399a/b/c and miR477, all showed down-regulation in response to heat stress (Additional file 1: Table S4). Similarly, down-regulation of miR159 was observed under heat in wheat [41] but an opposite trend was found in switchgrass [40]. All the discrepancies may result from the species-specific miRNA levels, or that miRNAs respond to heat stress only in specific tissues or at specific developmental stages.

### MiRNA-mediated pathways involved in banana temperature stress response

Based on miRNA expression profiling and dagradome combined with qRT-PCR validation, we found that several miRNA-target pairs might have played important roles in the regulation of banana temperature stress response. SPL transcription factors, which are unique to plants, are involved in many important biological events [45]. Most members of the SPL family are targets of miR156 and recent evidence showed that miR156 can down-regulate *SPL* expression by either mRNA cleavage or translational repression [46]. In banana, *SPL* genes can also be targeted by miR535 and our data confirmed the anti-correlation between miR156/miR535 and their corresponding target *SPL* genes (Fig. 4a). Moreover, an inverse expression pattern of the two tested *SPL* genes was observed at the later stage of storage, suggesting that *SPL* transcript accumulation may be balanced via the co-regulation of miR156 and miR535.

Another well anti-correlated module in banana was miR159 targeting PCF/TCP transcription factor. Upon cold treatment, miR159 gradually accumulated and remained at a higher level compared to the control. Accordingly, its *PCF* target gradually became suppressed as the injury of cold stress aggravated. Such pattern was opposite in the heat-treated sample (Fig. 4a). In rice, two miR319-targeted *PCF* genes (*OsPCF5* and *OsPCF8*) have been characterized as negative regulators of cold tolerance [47], which seemed to be different from our observation. Moreover, an earlier study [48] proposed that in *Arabidopsis*, *TCP*s are normally not targeted by miR159. However, our data showed that a banana *PCF* gene was targeted by miR159. In addition, we did not identify any miR319 variants in banana. These results implicated that banana may possess a functional specialization of miR159/miR319 different from *Arabidopsis*. In banana, without miR319, miR159 may have evolved to exert its regulation on *PCF*.

Besides the above mentioned miRNAs, we also found other miRNAs having differential responses to temperature stress. For example, miR397 showed a significant induction after heat stress treatment. Previous studies have shown that miR397 targets laccase (*LAC*) in rice, *Arabidopsis*, Populus and litchi [49, 50]. In banana, at least three annotated laccase genes were predicted as miR397 targets. We suggest that the downregulation of *LAC* was mediated by the induction of miR397, which might be involved in the thermotolerance through protection from oxidative damage. However, there has been no direct evidence for the relationship between oxidative damage and ripening disorder upon heat stress in banana. On the contrary, both miR398 and miR399 showed downregulation in response to heat. Target prediction identified their targets encoding putative cytochrome c oxidase subunit and ubiquitin-related modifier 1 homolog, respectively. MiR398 and its target sites on Cu/Zn superoxide dismutase genes (*CSD*s) mRNA are conserved in dicots and monocots [49, 51], with an important role in the oxidative stress tolerance in plants [51]. MiR399 has been reported to be responsive to phosphate starvation in *Arabidopsis* via targeting genes coding for putative ubiquitin-conjugating enzymes [52]. Under heat stress, banana fruit would consume more energy, so repressing of these two miRNAs would accumulate their target genes to adapt to the nutrient deficiency.

## Conclusions

Altogether, our work delivered new insights into the role of miRNAs in the temperature stress response of banana fruit. Most targets of temperature-responsive miRNAs in banana were transcription factors including a large group of target genes involved in auxin signaling, and other functional genes associated with redox/nutrient homeostasis. Emerging studies have demonstrated the utility of miRNAs for genetic improvement in crops [53, 54], so the characterized candidate miRNAs in our study may serve as markers in breeding programs or tools for biotechnological approaches for improving temperature stress tolerance of banana.

## Methods

### Plant materials and sample collection

Mature-green ‘Brazilian’ banana fruit (*Musa* spp. AAA group, cv. Cavendish) were harvested from a plantation located in Gaozhou County, Guandong province. Fruits were harvested at 80% of maturity and selected for uniformity and free of defects. All fruits were soaked in 0.2% (*w*/*v*) Sporgon solution (Bayer, Germany) for 5 min to eliminate potential microbes and air dried. Based on our previous trials, three storage temperatures were set in this study: control at 23 °C, cold stress temperature at 6 °C and heat stress temperature at 35 °C. For each temperature treatment, 120 fruit fingers were selected, packed in 0.025-mm-thick polyethylene bags, and divided into three groups of forty fingers each as biological replicates. Peel samples from control, cold- and heat-stressed fruit were collected 0, 2, 4, 6 and 8 d after treatment. All the samples were immediately frozen in liquid nitrogen and stored at − 80 °C until use.

### Observation of fruit appearance and firmness measurement under temperature stress

At each sampling time, photos were taken for fruit stored at different temperatures, which could represent a typical state. For each treatment, fruit firmness was measured at three equidistant points around the middle position on three unpeeled (peel firmness) and three peeled (pulp firmness) fruit fingers, respectively.

### Small RNA library construction and sequencing

Peel samples from control, cold- and heat-stressed fruits were collected on the 5^th^ day of storage, when the typical injury symptoms appeared. Two replicate total RNA from different samples was extracted using Trizol reagent (Invitrogen, CA, USA) following the manufacturer’s procedure. The total RNA quantity and purity were evaluated by Agilent Bioanalyzer 2100 and RNA 6000 Nano LabChip Kit (Agilent, CA, USA) with RIN number > 7.0. Approximately 1 μg of total RNA was used to prepare small RNA library according to protocol of TruSeq Small RNA Sample Prep Kits (Illumina, San Diego, USA). Then the single-end sequencing (36 bp) was performed on an Illumina Hiseq2000 at the LC-BIO (Hangzhou, China) following the vendor’s recommended protocol.

### Small RNA data analysis

Briefly, the raw reads were subjected to the Illumina pipeline filter (Solexa 0.3), and then the dataset was further processed with an in-house program, ACGT101-miR (LC Sciences, Houston, Texas, USA) to remove adaptor, low complexity, common RNA families (rRNA, tRNA, snRNA, snoRNA) and repeats. Subsequently, unique sequences with length of 18~25-nt were mapped to specific species precursors in miRBase 21 by BLAST search to identify known and novel 5p- and 3p-derived miRNAs. Length variation at both 3′ and 5′ ends and one mismatch inside of the sequence were allowed in the alignment. Unique sequences mapping to specific species mature miRNAs in hairpin arms were identified as known miRNAs, while unique sequences mapping to the other arm of known specific species precursor hairpin opposite to the annotated mature miRNA-containing arm were considered to be novel 5p- or 3p-derived miRNA candidates and the mapped pre-miRNAs were further compared against the specific species genomes using BLAST to determine their genomic locations.

The remaining unmapped sequences were compared against the banana genome (*Musa acuminata* DH-Pahang v1, banana-genome.cirad.fr) using BLAST to identify novel banana-specific miRNAs. The hairpin RNA structures containing sequences were predicated from the flanking 120-nt sequences using RNAfold software (http://rna.tbi.univie.ac.at/cgi-bin/RNAWebSuite/RNAfold.cgi) [55]. The criteria for secondary hairpin structure prediction were: (1) number of nucleotides in one bulge in stem (≤12). (2) number of base pairs in the stem region of the predicted hairpin (≥16). (3) cutoff of free energy (dG < − 35 kcal/mol). (4) length of hairpin (up and down stems + terminal loop ≥50). (5) length of hairpin loop (≤200). (6) number of nucleotides in one bulge in mature region (≤4). (7) number of biased errors in one bulge in mature region (≤2). (8) number of biased bulges in mature region (≤2). (9) number of errors in mature region (≤4). (10) number of base pairs in the mature region of the predicted hairpin (≥12). (11) percent of mature in stem (≥80). Based on the criteria, only 21~22-nt sRNAs with a good hairpin structure and a miRNA/miRNA* duplex accounting for more than 75% reads matching to the precursor locus were considered as potential banana-specific miRNAs.

### Degradome library construction, sequencing and data analysis

Mixed total RNAs were used, with equal amounts from all banana fruit at different stages of storage and also under temperature stress. Approximately 20 μg of total RNA was used to prepare a degradome library with following steps: (1) Biotinylated Random Primers (BRPs) were used to incubate with ~ 150 ng of poly (A)^+^ RNA (2) RNA fragments annealed with BRPs were captured by Strapavidin. (3) 5′ adaptor ligation to only those RNAs containing 5′ monophosphates. (4) Reverse transcription and PCR. (5) Libraries were sequenced using the 5′ adaptor only, resulting in the sequencing of the first 36 nucleotides of the inserts that represented the 5′ ends of the original RNAs. Then the single-end sequencing (36 bp) was performed on an Illumina Hiseq2500 at the LC-BIO (Hangzhou, China) following the vendor’s recommended protocol. Raw sequencing reads were obtained using Illumina’s Pipeline v1.5 software following sequencing image analysis by Firecrest Module and base-calling by Bustard Module. The extracted sequencing reads were used in the standard data analysis. A public software package, CleaveLand 3.0 was used for analyzing sequencing data generated, with alignment score (AS) threshold set to 4. Reads at the cleavage site were normalized to transcripts per billion (tpb).

### Analysis of differential expressed miRNAs under temperature stress

Total number of reads perfectly matching the banana genome in a given library was used for the normalization of read abundance, which was denoted as RPM (reads per million genome-matched reads). The miRNA differential expression based on normalized deep-sequencing counts was analyzed by Student *t* test based on the experimental design. The significance threshold was set to be *p* < 0.01 with fold changes (stress reads/control reads) > 1.2-fold for identifying differentially expressed miRNAs upon cold or heat stress. TBtools software was used for the construction of the heat map [56].

### *PHAS* loci identification

PhasiRNAs are produced with the ‘phasing’ pattern and this pattern can be used to identify phasiRNA-generating loci (*PHAS* loci), based on reference-aligned sRNA-seq data. After mapping sRNA reads to the reference genome, unique sRNAs were denoted with their matching coordinates. Two-nucleotide offset was added for sRNA matching to the antisense strand due to the 2-nt overhang at the 3′ end of the sRNA duplex. A genome-wide search was performed using a nine-cycle sliding window with each shift of three cycles, and a window was reported when ≥10 distinct sRNA reads fell into it; more than half of the mapped distinct sRNAs were 21-nt in length, and with ≥3 distinct reads in a certain register. All reported windows with overlapping region were then combined into a single longer window and a *P*-value was calculated for each window [31, 57]. In addition to a *P*-value threshold of 0.001, additional criteria were applied to ensure that only highly confident *PHAS* loci were identified: (1) the length of phasiRNA-producing region ≥100 bp. (2) over 30% of the siRNAs from a given *PHAS* locus were produced in phase. (3) the sRNA genomic matches were ≤ 10. The *PHAS* loci identification was done separately for each sample, and results were merged to a non-redundant list based on the genomic coordinates. Next, the potential miRNA triggers of *PHAS* loci were predicted using ‘reverse computation’ method as described in [31].

### RNA gel blot

Total RNA was extracted from peel samples of control, cold- and heat-stressed fruit with Plant RNA Isolation Reagent**®** (Invitrogen). For gel blot, 20 μg of total RNA from banana fruit samples was separated on a 15% denaturing polyacrylamide gel and transferred to Amersham Hybond™-NX membranes (GE Healthcare, Waukesha, WI, USA). RNA was cross linked using EDC (N-(3-dimethylaminopropyl)-N′-ethyl-carbodiimide hydrochloride (Sigma, St Louis, MO, USA). The probes of 21~22-nt DNA oligonucleotides (Additional file 1: Table S7) reverse complementary to banana miRNA candidates were labeled with γ**-**[^32^p]-ATP by T4 polynucleotide kinase (NEB, Ipswich, MA, USA). Non-incorporated nucleotides were removed using microspin G-25 column (GE Healthcare, Buckinghamshire, UK). Blots were also probed with a DNA probe complementary to U6 to confirm uniform loading. The prepared membrane filters were hybridized at 42 °C overnight, then washed twice at 55 °C with washing buffer containing 2 × SSC and 2% SDS. Membranes were then exposed to phosphorscreens and scanned with a Typhoon TRIO Variable Mode Imager (GE Healthcare). Membrane exposure time was adjusted, dependent on signal intensity.

### RLM-5’-race

Five micrograms of total RNA isolated from banana peel was used for ligating 5’ RNA adaptors at 37 °C and then reverse transcription at 42 °C for nested PCR for 5′ RLM-RACE, following the manufacturer’s instructions for FirstChoice RLM-RACE Kit (Ambion, Austin, TX, USA). Gene-specific primers (Additional file 1: Table S7) were designed to conduct nested PCRs, and PCR products were gel purified, cloned into the pMD18-T vector (TaKaRa) and sequenced.

### Quantitative real-time PCR validation of differentially expressed miRNAs and their target genes

Quantitative real-time PCR was carried out using the same RNA samples used for gel blot analysis. Total RNA (1 μg) was treated with DNase I and reverse transcribed with PrimeScript™ RT Kit (Takara), according to the manufacturer’s instructions but using specific stem-loop RT primers for miRNAs and oligo-dT primer for target mRNAs (Additional file 1: Table S7). Real-time PCR analysis was carried out using SYBR**®** *Premix Ex* Taq™ II (TaKaRa) on an ABI 7500 PCR System (Applied Biosystems), according to the standard protocol. The analysis was performed using three independent cDNA preparations and triplicate PCR reactions. The relative expression was calculated using 2^-(∆∆Ct)^ method with 5 s ribosomal RNA (5 s rRNA) and *Actin* as references for miRNAs and target genes, respectively.

## Additional file

Additional file 1: **Table S1.** Statistics of sRNA sequences from banana. **Table S2.** Summary of known miRNAs in banana. **Table S3.** Summary of species-specific miRNAs in banana. **Table S4.** Summary of differentially expressed miRNAs in respond to temperature stress in banana. **Table S5.** Target genes of known miRNAs in banana. **Table S6.** *PHAS* loci in banana. **Table S7.** Probes for RNA gel blot and primers for qPCR and RLM-5’-RACE. (XLSX 83 kb)

## Abbreviations

AGO: Argonaute
AS: Alignment score
DCL: Dicer-like
miRNAs: MicroRNAs
nt: Nucleotide
*PHAS* loci: Phased siRNA-generating loci
phasiRNAs: Phased siRNAs
qRT-PCR: Quantitative reverse transcription real-time PCR
RISC: RNA-induced silencing complex
RLM-5’-RACE: RNA ligase-mediated rapid amplification of cDNA 5′ end
RPM: Reads per million genome-matched reads
sRNAs: Small RNAs
tpb: Transcripts per billion
